# NeuroD1 administration ameliorated neuroinflammation and boosted neurogenesis in a mouse model of subarachnoid hemorrhage

**DOI:** 10.1186/s12974-023-02949-w

**Published:** 2023-11-12

**Authors:** Ping Chen, Xue-Yan Liu, Mou-Hui Lin, Yu-Xi Li, De-Zhi Kang, Zu-Cheng Ye, Qing-Song Lin

**Affiliations:** 1 Department of Anesthesiology, Anesthesiology Research Institute, The First Affiliated Hospital of Fujian Medical University, Fuzhou, Fujian China; 2 Key Laboratory of Brain Aging and Neurodegenerative Diseases, The School of Basic Medical Sciences, Fujian Medical University, Fuzhou, Fujian China; 3 Department of Medicinal Chemistry, School of Pharmacy, Fujian Medical University, Fuzhou, 350122 Fujian China; 4 Department of Neurosurgery, Neurosurgery Research Institute, First Affiliated Hospital of Fujian Medical University, No. 20 Chazhong Rd, Taijiang District, Fuzhou, 350005 Fujian China; 5 Department of Neurosurgery, Binhai Branch of National Regional Medical Center, First Affiliated Hospital of Fujian Medical University, Fuzhou, 350209 Fujian China; 6 Fujian Provincial Institutes of Brain Disorders and Brain Sciences, First Affiliated Hospital of Fujian Medical University, Fuzhou, 350005 Fujian China; 7 Fujian Provincial Clinical Research Center for Neurological Diseases, First Affiliated Hospital of Fujian Medical University, Fuzhou, 350005 Fujian China; 8 Fujian Provincial Key Laboratory of Precision Medicine for Cancer, First Affiliated Hospital of Fujian Medical University, Fuzhou, 350005 Fujian China

**Keywords:** NeuroD1, Astrocyte, Neuroinflammation, Neurogenesis, Neurocognitive function, Subarachnoid hemorrhage

## Abstract

**Background:**

Subarachnoid hemorrhage (SAH) causes significant long-term neurocognitive dysfunction, which is associated with hippocampal neuroinflammation. Growing evidences have shown that astrocytes played a significant role in mediating neuroinflammation. Recently, in vivo reprogramming of astrocytes to neurons by NeuroD1 or PTBP1 administration has generated a lot of interests and controversies. While the debates centered on the source of neurogenesis, no attention has been paid to the changes of the astrocytes-mediated neuroinflammation and its impact on endogenous neurogenesis after NeuroD1 administration.

**Methods:**

80 adult male C57BL/6 mice were used in this study. SAH was established by pre-chiasmatic injection of 100 μl blood. AAV–NeuroD1–GFP virus was injected to the hippocampus 3 day post-SAH. Neurocognitive function, brain water content, in vivo electrophysiology, Golgi staining, western blot and immunofluorescent staining were assessed at day 14 post-virus injection.

**Results:**

NeuroD1 administration markedly attenuated reactive astrocytes-mediated neuroinflammation by reversing neurotoxic A1 astrocytes transformation, decreasing the secretion of neuroinflammatory cytokines, and reducing the activation of harmful microglia. NeuroD1 treatment significantly reversed the brain–blood barrier impairment and promoted the release of neurotrophic factors pleiotrophin (PTN), all of which contributed to the improvement of cellular microenvironment and made it more suitable for neurogenesis. Interestingly, besides neurogenesis in the hippocampus from cells transfected with NeuroD1 at the early phase of SAH, NeuroD1 administration significantly boosted the endogenous neurogenesis at the late phase of SAH, which likely benefited from the improvement of the neuroinflammatory microenvironment. Functionally, NeuroD1 treatment significantly alleviated neurocognitive dysfunction impaired by SAH.

**Conclusions:**

NeuroD1 significantly promoted neurofunctional recovery by attenuating reactive astrocytes-mediated neuroinflammation and boosting neurogenesis decimated by SAH. Specifically, NeuroD1 efficiently converted transfected cells, most likely astrocytes, to neurons at the early phase of SAH, suppressed astrocytes-mediated neuroinflammation and boosted endogenous neurogenesis at the late phase of SAH.

**Supplementary Information:**

The online version contains supplementary material available at 10.1186/s12974-023-02949-w.

## Introduction

Subarachnoid hemorrhage (SAH) leads to substantial long-term neurocognitive deficits, which is associated with neuroinflammation in the hippocampus [1]. Contrary to the previous notion that microglia was the sole mediator of neuroinflammation, growing evidences have shown that astrocyte had a major role in mediating neuroinflammation in brain injury and diseases [2–4]. Astrocyte can be highly responsive and undergo pronounced transformation to become “reactive astrocytes” [5]. Reactive astrocyte can shift toward a harmful, pro-inflammatory A1 phenotype or a beneficial, anti-inflammatory A2 phenotype[6]. Astrocyte also plays a crucial role in brain–blood barrier (BBB) integrity and maintains suitable microenvironment for synaptic function [7]. SAH has been reported to induce significant A1 astrocyte transformation, widely spread neuroinflammation, compromise BBB integrity and injure neuronal function [8]. Tilting A1 astrocyte toward alternative A2 astrocyte became a choice for attenuating brain injury.

Recently, in vivo reprogramming technology has emerged to be a powerful way for neural regeneration, and several transcription factors have been identified to efficiently convert astrocyte into neuron [9–11]. Among these, NeuroD1 is one of the most popular transcription factors studied [12]. By injecting NeuroD1 alone, reactive astrocyte was reported to be directly converted into functional neuron, which significantly attenuated neuronal deficit in ischemic brain injury and Alzheimer’s Disease [10, 13]. Since previous studies mainly focused on the mechanism of NeuroD1-mediated astrocyte reprogramming, the role of NeuroD1 in regulating reactive astrocyte-mediated neuroinflammation remained largely unknown. Moreover, whether NeuroD1 really induced astrocyte reprogramming remained debatable, since it has been argued that AAV–NeuroD1 may act by awakening the neural stem cell (NSC) niche to originate the neurons, but not local cortical astrocytes [14]. NSC niche is a well-established concept, which emphasizes the importance of microenvironment on maintaining and rejuvenating neural stem cells [15]. Whether NeuroD1 attenuates reactive astrocyte-mediated neuroinflammation and provides a suitable microenvironment for endogenous neurogenesis remains undefined.

In this study, we found that hippocampus subgranular zone (SGZ) happened to be a region most severely affected by SAH, it provided an ideal model for studying the effect of NeuroD1 on astrocyte-mediated neuroinflammation and endogenous neurogenesis. We, therefore, established a mouse model of SAH and injected AAV–gfaABC1D–NeuroD1–GFP virus into the hippocampus to investigate the role of NeuroD1 in regulating reactive astrocytes-mediated neuroinflammation. We found that at the early stage after SAH and NeuroD1 administration at 14dpi, NeuroD1 mainly converted transfected cells into neurons, accompanied with significantly attenuated neuroinflammation and recovery from BBB disruption, the later created a more suitable microenvironment for endogenous neurogenesis at 23dpi. It has been well-established that neurons originated from endogenous adult hippocampal neurogenesis (AHN) can well incorporate into existing neural network and is key to learning and memory. Above all, together with NeuroD1-initiated conversion, NeuroD1-rejuvenated microenvironment reboot AHN and significantly repaired the neural circuit and promoted neurofunctional recovery after SAH.

## Materials and methods

### Animals and SAH model

Adult C57BL/6 mice (3–4 months, male) were used in this study. The mice were kept under controlled temperature and humidity conditions with a 12-h light/dark cycle and free access to water and food. All experimental protocols were approved by the Animal Care and Use Committee of Fujian Medical University and were in accordance with the Institutional Animal Care and Use Committee (IACUC) of Loma Linda University.

SAH model was established by pre-chiasmatic injection of blood as previously reported [1]. Briefly, mice were performed avertin anesthesia (1.2% avertin, 20 ml/kg) and then fixed in a stereotactic apparatus. A midline incision was cut and the transparent skull was exposed. A burr hole in the skull 4.5 mm anterior to the bregma was drilled with a caudal angel of 40° using a 0.9-mm drill. A total of 100 ul blood withdrawn from C57BL/6 WT or eGFP blood donor was injected with a needle through the hole at an angel of 40° until reaching the skull base for over 15 s. The needle was kept in place for 10 min to avoid backflow. After surgery, mice were kept individually in heated cages for recovery.

### Viral injection

pAV–GfaABC1D–NeuroD1–P2A–GFP (ND1–GFP) and pAV–GfaABC1D–P2A–GFP (GFP) virus were purchased from Vigenebio (Shandong, China). At 3 days after SAH induction when astrocytes became reactive, mice were randomly subjected to either NeuroD1 or control virus injection into the same site as previously reported [16]. Specifically, a total of 1.5 μl AAV was injected into the hippocampus using a 5 μl micro-syringe and a 34 Gauge needle (Hamilton) [16]. The viral injective rate was controlled at 0.15 μl/min, with the needle gradually moved up at a speed of 0.1 mm/min. The needle was maintained in place for additional 3 min. After injection, mice were kept individually in heated cages for recovery.

### In vivo electrophysiology

As previous research [17], mice were anesthetized with 1.2% avertin (20 ml/kg) for collecting data related to spike transmission. The skull was sterilized with hydrogen peroxide and saline and ground wires were placed intracranially over the cerebellum at 7 dpi. A craniotomy was carried out at AP: 2.06 mm, ML: 1.6 mm (right hemisphere) from the midline. The dura was opened, and the microelectrode wire was implanted into the cortex. The probe and custom driver were cemented to the skull with Quick Adhesive Cement. the craniotomy was covered with a mixture of quick self-curing acrylic resin (Re-fine Bright, China). After surgery, mice were housed individually on a 12/12 h day/night schedule.

After 1 week, animals were recorded for data collection. The microelectrode array (3 × 5, inter-wire interval, 300 μm; wire diameter, 33 μm) was implanted in the dentate gyrus of the hippocampus at a depth of 1.8 ± 0.2 mm from the skull surface). Data were analyzed at 30 kHz using a brain signal acquisition system (NeuroStudio, Jiangsu Brain medical technology company, China). After the experiments, the brain was postfixed for verifying the appropriate location of the electrodes in the target region.

### Immunofluorescence staining

Immunofluorescence staining was performed as previously reported [16]. Briefly, 30 μm brain sections were cut using a cryo-stat microtomes (Leica). Appropriate slices were blocked with blocking buffer, and then incubated with primary antibodies at 4 °C overnight. The primary antibodies incubated were as follows: GFAP (NB300-141, NOVUS), GFAP (C9205, sigma), NeuroD1(311A3A12, invitrogen), DCX (ab207175, abcam), NeuN (ab104224, abcam), LY6C (ab15627, abcam), Nestin (33475, CST), SOX2 (ab97959, abcam), C3 (ab11887, abcam), iNOS (sc-7271, Santa Cruz), Iba-1 (019-19741, wako), C1q (abcam, ab182451), a-synuclein (Syn, ab32127,abcam), PSD95 (MAB1596, sigma). After washing with PBS, the brain sections were incubated with relative secondary antibodies for 2 h at room temperature. All slices were adhered to slide-glass and photographed under a fluorescence microscope (Leica Microsystems, Germany).

### Morphology analysis of astrocytes

The morphologic alteration of astrocytes was assessed using ImageJ software (version 2.9.0/1.53t) combined with the plugins of NeuronJ and Sholl analysis as previous report [7]. The representative astrocytes immunolabeled by GFAP were reconstructed and projected into outline and trace patterns for the observation of cellular morphology. The branch count, cellular perimeter, soma volume and sum dendritic length of astrocytes were measured by ImageJ and compared within groups.

### Western blot

Western blot was conducted as previously described [18]. In brief, the relative hippocampus tissue was harvested to extract proteins, and protein concentration was measured using a BCA kit (Beyotime Biotechnology). Equal amounts of protein sample were run on an SDS–PAGE gel and then transferred to a nitrocellulose membrane. The membranes were blocked at room temperature and incubated at 4 °C overnight with the following primary antibodies: C3 (ab200999, abcam), DCX (ab207175, abcam), Neuron (ab104224, abcam), PTX3 (13971-AP, Proteintech), PSD95 (P246, sigma), pleiotrophin (PTN, ab79411, abcam), Tubulin (10068-1-AP, Proteintech), a-synuclein (Syn, ab32127,abcam), TNF-a (#11948, CST), IL-18(#57058, CST), Actin (#3700, CST), GAPDH (10494-1-AP, Proteintech). An ECL reagent was used to visualize the specific bands. The relative density of each immunoblot band was analyzed using ImageJ software.

### Golgi staining

The hippocampal dendritic spines were measured using the Hito Golgi-Cox OptimStainTM kit (USA) as previously reported [19]. Briefly, brains were harvested at 14 dpi and incubated in impregnation solutions for 2 weeks in the dark at room temperature. The brains were transferred to solution 3 for 1 week and 150 mm thick coronal sections were cut at around 1.9 mm and -2.7 mm from bregma on a vibratome. The tissues were mounted to slides and stained. Photographs were taken with a Zeiss Axio Imager Z2 microscope. Neurons in the hippocampus were randomly selected from each group and images were taken with an oil objective.

### PI staining

PI staining was performed to assess membrane permeability as previously reported [20]. Briefly, PI (Sigma) was injected intraperitoneally (3 mg/kg) 2 h before sacrificing the mice. Frozen brain sections were obtained and counterstained with 4’,6-diamidino-2-phenylindole, and then immediately photographed.

### Behavioral performance

The novel object recognition was conducted at 11–14 day post-virus injection (dpi) as previously reported [1]. After 3 days of animal adaptation in the open field box for 10 min, two identical objects were placed in the box on 14 dpi. The animal was placed in the middle of the same objects and allowed to explore for 5 min. 90 min late, one of the objects was replaced with a novel object. The animal was allowed to explore for 5 min. The time exploring objects were recorded by ANY-maze software. The NOR discrimination index was evaluated as (the time on the new object/the total exploration time) *100%.

Morris water maze was performed to assess the learning and memory function [21]. Briefly, Morris water maze included a black circular pool, which filled with warm (23–25 °C) water. From day 9 to day 13 after AAV injection, mice were trained in the standard Morris water maze with a platform (1.5 cm beneath the water surface), the escape latency to find the platform was recorded. Each mouse was placed into the water of every quadrant and a maximum of 60 s was permitted to search the platform. If animals failed to find the platform in 60 s, they were guided to the platform and kept on the platform for 10 s. In the probe trial on day 14 after AAV injection, the platform was removed, and the number of platforms crossing and the distance to the platform were recorded.

### Statistical analysis

The statistical analyses were performed using GraphPad Prism software. Data were presented as mean ± standard deviation. Student t test was used to analyze difference between the two groups. One-way ANOVA was applied for multiple comparisons among groups, followed by Tukey’s multiple comparison test. *P* < 0.05 was considered as statistical significance.

## Results

A total of 80 mice were used in this study. Blood clots were accumulated mainly around the circle of Willis and the ventral surface of brainstem after SAH induction, no blood clot was observed in the sham group (Additional file 1: Fig. S1). SAH caused an overall mortality rate of 8.75% (7/80), and no mouse died in the sham group. PI staining showed that SAH induced significant neuronal death and the PI-positive cells mainly distributed around the SGZ (Additional file 1: Fig. S2). Immunofluorescent images revealed that SAH significantly induced astrocyte reactivation in the hippocampus at 3 days after SAH, as indicated by the large increase in GFAP immunoreactivity (Additional file 1: Fig. S3).

To target the reactive astrocytes, astrocytic promoter GFAP was used to drive the expression of NeuroD1 and reporter GFP in astrocytes specifically. ND1–GFP or GFP virus was injected into the hippocampus at 3 days after SAH induction, and then the relative tissue was harvested at 3 dpi, 7 dpi, 14 dpi as illustrated in Additional file 1: Fig. S4A. Immunofluorescent images showed that ND1–GFP efficiently increased the expression of NeuroD1 in the hippocampus compared with that in the GFP group at each relative timepoint (Additional file 1: Fig. S4B–D).

### NeuroD1-attenuated reactive astrocyte-mediated neuroinflammation after SAH

**Fig. 1:**
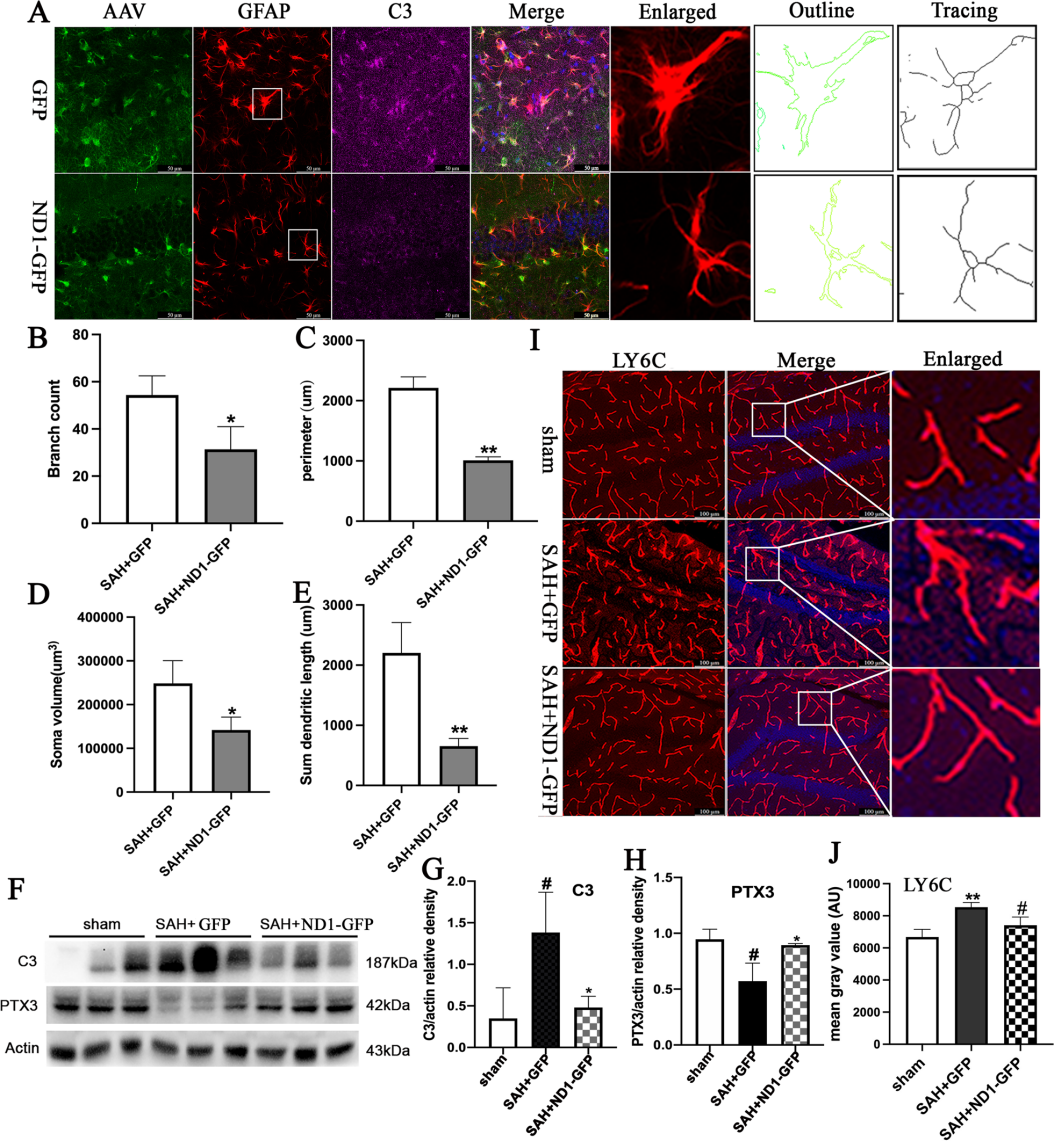
ND1–GFP-attenuated reactive astrocyte-mediated neuroinflammation following SAH. **A** Representative immunofluorescent images showed the C3-labeled GFAP-positive astrocytes and the morphological analysis of astrocytes (enlarged) in the hippocampus of the ND1–GFP group and the GFP group at 14 dpi. *N* = 6 per group. Scale bar, 50um. **B** Dendritic branch count of the astrocytes. **C** Soma volume measurement of the astrocytes. **D** Cellular perimeter analysis of the astrocytes. **E** Sum dendritic length of the astrocytes. **F**–**H** Representative western blot and quantification analysis showed the protein levels of C3 and PTX3 in the hippocampus from different groups after SAH. **I**, **J** Representative images and quantification analysis of LY6C staining showed the blood vessel morphology in different groups after SAH. Scale bars = 100 μm. ^#^ *P* < 0.05 indicated comparison with Sham group; *P < 0.05 indicated comparison with SAH + GFP group; *n* = 6 per group

Reactive astrocyte acts as a two-edge sword which may induce harmful effect by secreting inflammatory cytokines or serve as a physical barrier to prevent spreading of injury[2]. We wondered what impact may occur to reactive astrocyte after NeuroD1 treatment. As illustrated in Additional file 1: Fig. S5A, the immunofluorescent images revealed that NeuroD1 treatment significantly ameliorated the activation of astrocyte in the hippocampus than that in the GFP group at 3dpi, 7dpi and 14dpi (Additional file 1: Fig. S5B–D). C3 was used to label neurotoxic A1 astrocytes[6], and immunofluorescent staining showed that NeuroD1 significantly decreased the number of C3-labeled GFAP-positive astrocytes than that in the GFP group at 14 dpi (Fig. 1A). Representative images of reactive astrocytes in the hippocampus were traced into outline and branch patterns, and the quantitative analysis showed that the branch count, cellular perimeter, soma volume and sum dendritic length of the reactive astrocytes were all significantly increased after SAH. However, NeuroD1 treatment markedly reversed all these parameters than those in the GFP group at 14 dpi (*P* < 0.05, Fig. 1B–E.

PTX3 was introduced as a molecular marker for neuroprotective A2 astrocytes[6], and western blot showed that PTX3 was significantly decreased after SAH. However, NeuroD1 treatment promisingly increased the protein level of PTX3 than that in the GFP group (*P* < 0.05, Fig. 1F, H). Furthermore, western blot showed that C3 was significantly increased after SAH, and NeuroD1 markedly decreased the C3 protein level compared with that in the GFP group at 14 dpi (*P* < 0.05, Fig. 1F, G).

Astrocyte can function by interacting with blood vessel and contributing to BBB to limit harmful stimulation from chemical and biological toxicity[7]. SAH can induce severe BBB impairment as reported in our previous studies[1, 22]. In this study, immunohistochemistry for LY6C and quantification analysis showed that SAH caused significant blood vessel swollen at 14 dpi, ND1–GFP treatment markedly reversed the hypertrophic blood vessels than those in the GFP group (Fg. 1I, J), which indicated that ND1–GFP treatment restored the leaky BBB following SAH.

**Fig. 2:**
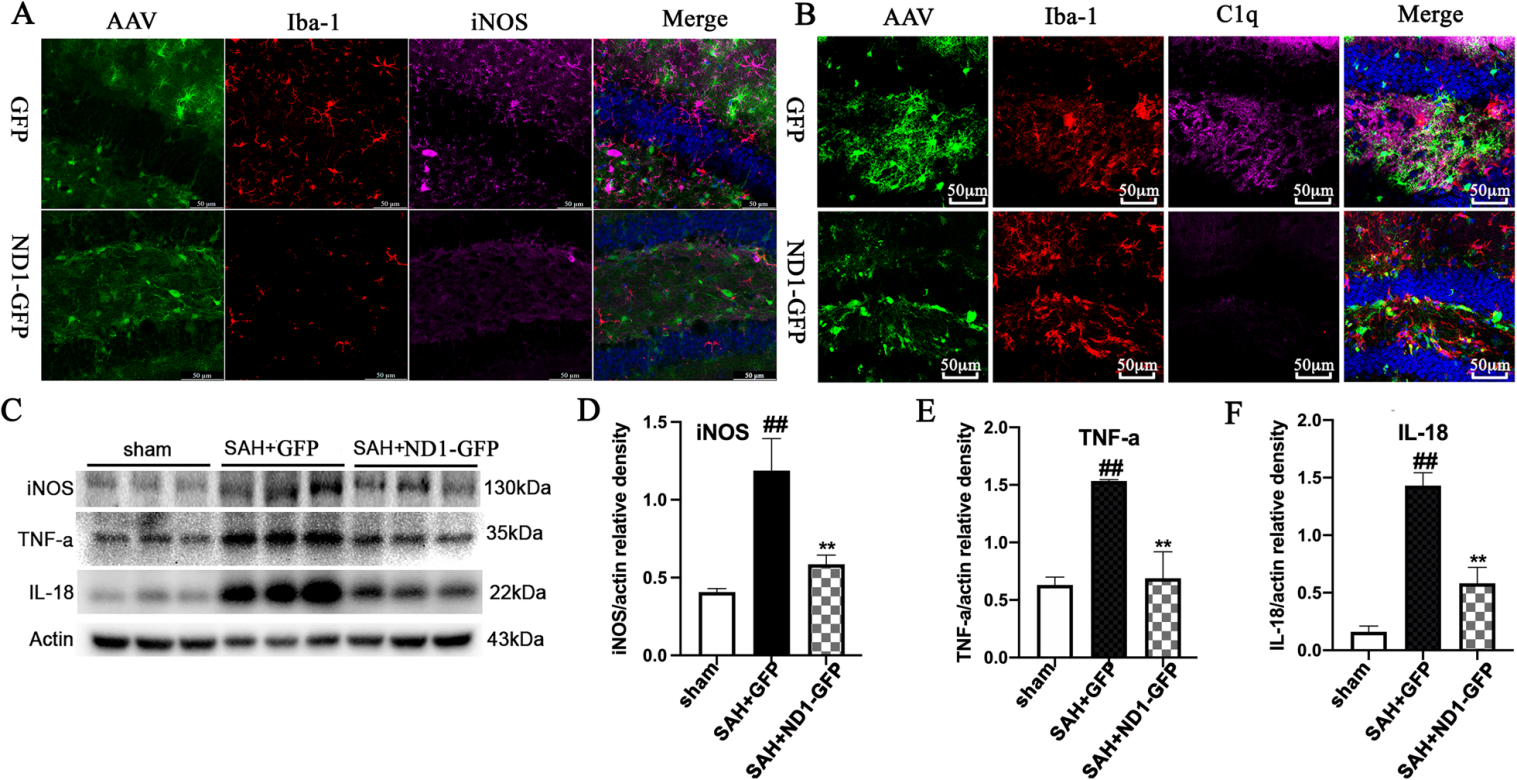
ND1–GFP reversed astrocyte-induced activation of neurotoxic microglia and reduced the inflammatory cytokines. **A** Representative immunofluorescent images revealed the activation of iNOS^+^Iba-1^+^ microglia in the hippocampus at 14 dpi after NeuroD1 treatment. *n* = 6 per group. Scale bar, 50 μm. **B** Immunofluorescent staining showed that the expression of C1q in mice with or without ND1–GFP treatment. **C**–**F** Representative western blot and quantification analysis showed the protein levels of iNOS, TNF-a and IL-18 in the hippocampus from different groups after SAH. ^#^*P* < 0.05 and ^##^ *P* < 0.01 indicated comparison with Sham group; *P < 0.05, **P < 0.01 indicated comparison with SAH + GFP group; *n* = 6 per group

A1 astrocyte can mediate neuroinflammation by activating neurotoxic microglia and releasing inflammatory cytokines [23]. iNOS was used to mark harmful microglia, and immunofluorescent results showed that injection of ND1–GFP markedly reduced the number of iNOS^+^Iba-1^+^ microglia than that in the GFP group (Fig. 2A). Notably, microglia contacting NeuroD1-infected astrocytes exhibited less amoeboid shape and more processes as compared to the microglia contacting AAV–GFP-infected astrocytes (Fig. 2A). Moreover, immunofluorescent images showed that ND1–GFP administration significantly reduced the amount of C1q than that in the GFP group (Fig. 2B). Furthermore, western blot showed that inflammatory cytokines iNOS, TNF-α and IL-18 were significantly increased after SAH induction, and NeuroD1 treatment strongly reduced the amount of iNOS, TNF-α and IL-18 than those in the GFP group (*P* < 0.05, Fig. 2C–F). Taken together, these results indicated that NeuroD1-attenuated reactive astrocyte-mediated neuroinflammation following SAH.

### NeuroD1 improved the microenvironment and boosted neurogenesis following SAH

**Fig. 3:**
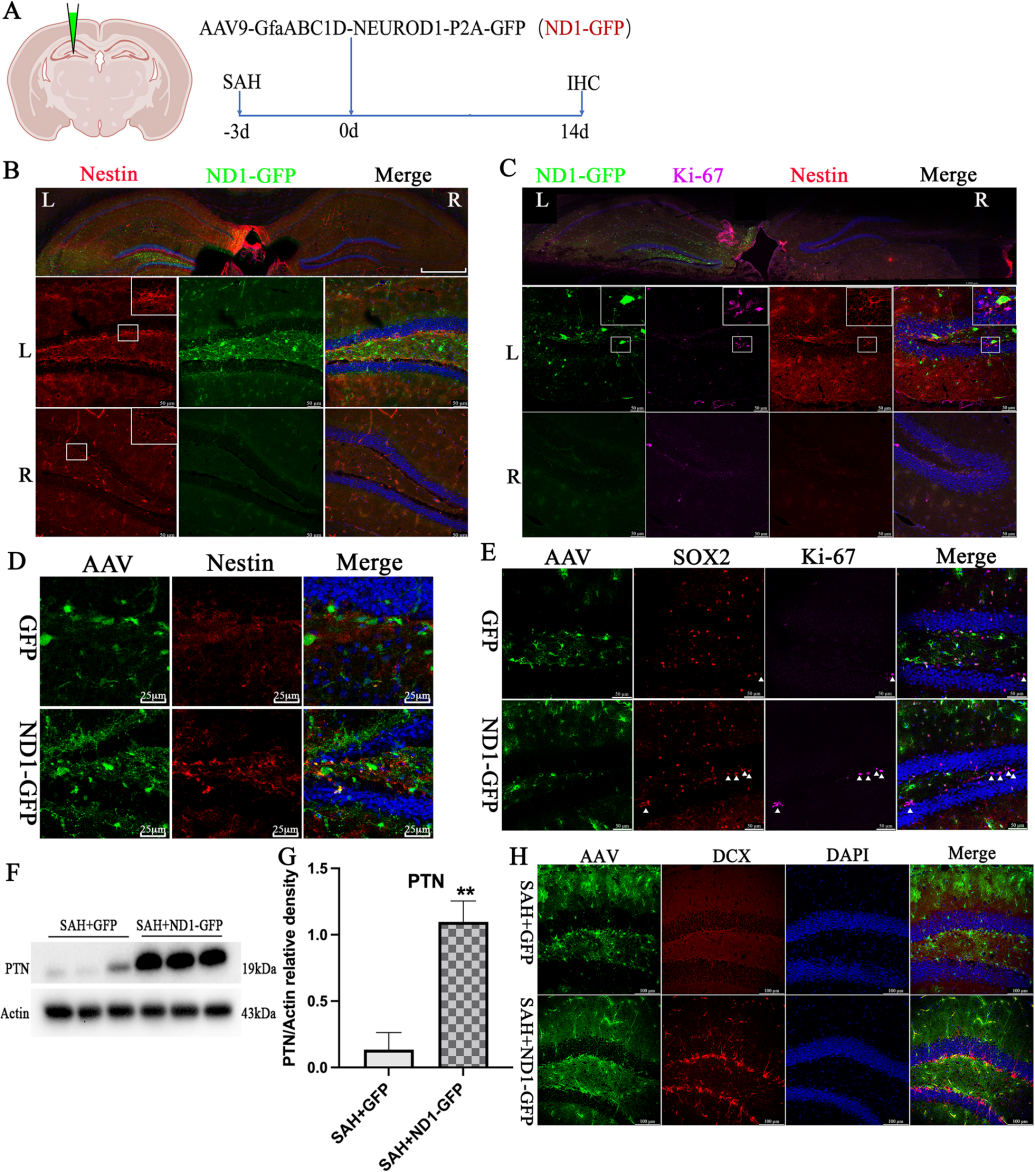
ND1–GFP improved the cellular microenvironment and boosted neurogenesis following SAH. **A** Schematic illustration of the experimental protocol. **B** Representative immunofluorescent image showed the whole brain immunostaining for Nestin, and the magnified image revealed that the intensity of Nestin was significantly higher in the ND1–GFP side (L) than that in the control GFP side (R) at 14 dpi. Scale bar: 500 μm for whole brain section 50 μm for the enlarged images. L, left. R, right. **C** Representative immunofluorescent image showed the whole brain immunostaining for Ki-67 and Nestin, and the enlarged images showed that ND1–GFP-boosted Nestin expression and promoted Ki-67 labeled cell proliferation in the subgranular zone known for neurogenesis. Scale bar: 500 μm for whole brain section 50 μm for the enlarged images. L, left. R, right. **D** Representative immunofluorescent image showed the expression of Nestin in mice with or without ND1–GFP treatment. Scale bar, 25 μm. **E** Representative immunofluorescent image revealed the number of SOX2^+^Ki-67^+^ cells in the hippocampus in mice with or without ND1–GFP treatment. Scale bar, 25 μm. **F**, **G** Representative western blot image and quantification analysis of PTN in the hippocampus at 14 dpi. *N* = 6. ^**^p < 0.01 indicated comparison with SAH + GFP group. **H** Representative immunofluorescent image showed the expression of DCX in the hippocampus at 14dpi after NeuroD1 treatment. *N* = 6. Scale bar: 100 μm

The SGZ is one of the most important NSC niches which is vulnerable to the neuroinflammatory microenvironment [24]. To determine the impact of SAH and subsequent NeuroD1 application on NSC niches, we tested Nestin, a molecular marker for NSC. ND1–GFP virus was injected to the left hippocampus, and the vehicle was injected to the right hippocampus of the same mouse at 3 days after SAH induction (Fig. 3A). Immunofluorescent images showed that the expression of Nestin was significantly higher in the ND1–GFP-injected side than that in the vehicle side at 14 dpi (Fig. 3B). Interestingly, ND1–GFP promisingly increased the number of Ki-67 positive cells over the control side, and Ki-67 positive cells were in a region with relatively higher Nestin expression levels (Fig. 3C). Furthermore, we conducted experiments that injected ND1–GFP virus and control GFP virus using separate mice, and the immunofluorescent images showed that ND1–GFP treatment significantly upregulated the expression of Nestin than that in the GFP group at 14 dpi (Fig. 3D). Moreover, SOX2, another molecular marker for NSC, was used to identify the cellular localization of Ki-67, and immunofluorescent images showed that ND1–GFP markedly increased the number of SOX2^+^Ki-67^+^ cells in the hippocampus than those in the GFP group at 14 dpi (Fig. 3E), suggesting that ND1–GFP altered the cellular microenvironment favoring the proliferation of NSCs in SGZ at 14 dpi.

Astrocyte-derived PTN can reduce pro-inflammatory signaling and is crucial for the maturation of newborn neurons [25, 26]. We wondered whether NeuroD1 treatment affected the secretion of PTN, and western blot revealed that the protein level of PTN was significantly higher in the ND1–GFP group than that in the GFP group at 14 dpi (*P* < 0.05, Fig. 3F, G). Furthermore, immunofluorescent images also showed that ND1–GFP injection significantly increased the number of DCX-positive neurons at SGZ than that in the GFP group at 14 dpi (Fig. 3H). Together, these results indicated that ND1–GFP treatment-boosted neurogenesis, which likely benefited from the improvement of neuroinflammatory microenvironment following SAH.

### NeuroD1-converted transfected cells into neurons at the early phase and boosted endogenous neurogenesis at the late phase of SAH

**Fig. 4:**
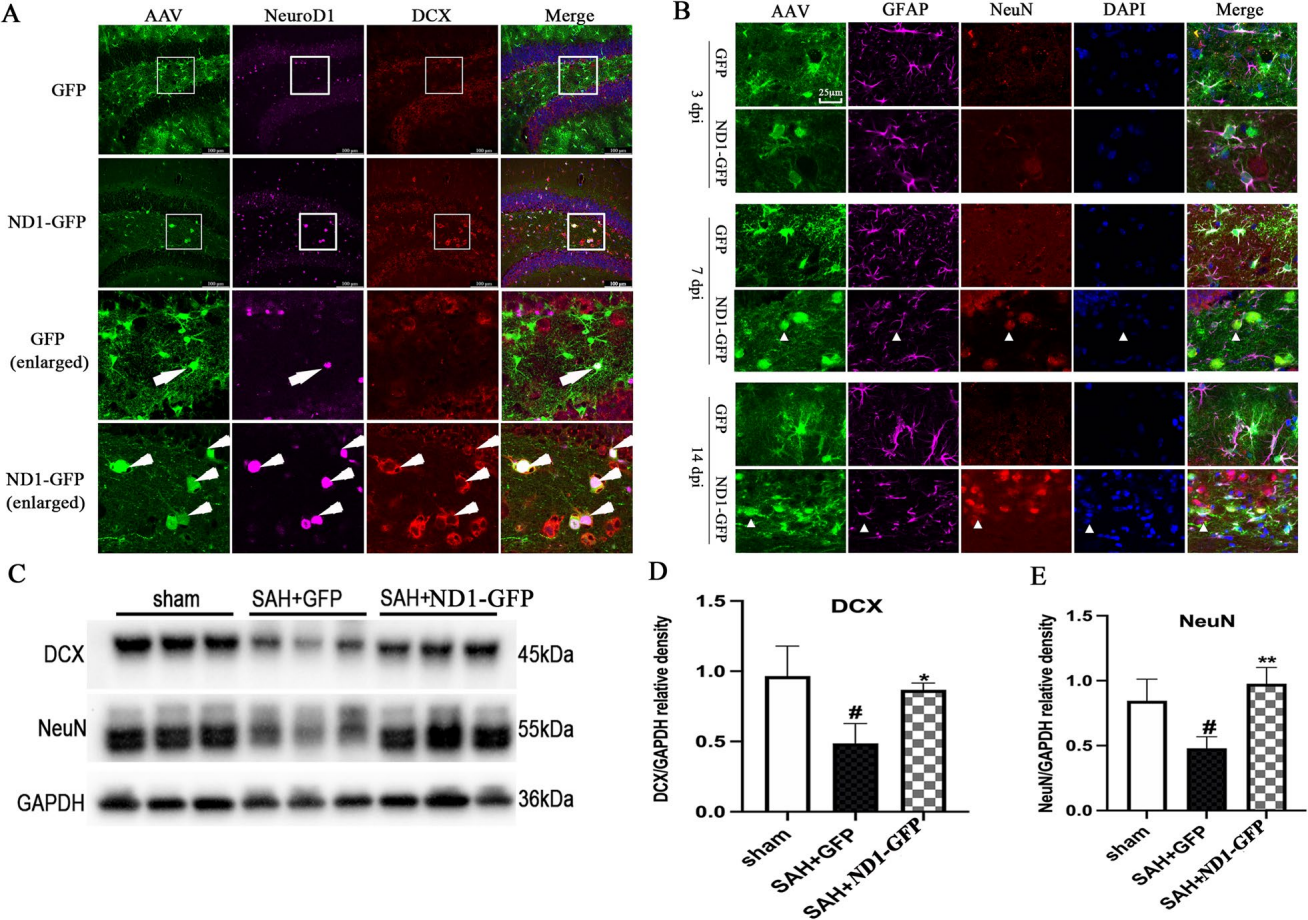
ND1–GFP efficiently converted astrocytes to neurons in the hippocampus after SAH. **A** Represent image showed the expression of DCX in the hippocampus at 14 dpi with or without ND1–GFP treatment. The white arrowhead in the enlarged images showed colocalization of GFP (green), NeuroD1 (purple) and DCX (red), representing a transitional stage in astrocyte to neuron conversion. Scale bar: 100 μm. **B** Represent immunofluorescent images showed the expression of NeuN in the hippocampus from 3 to 14 dpi. The white arrowhead in the enlarged images showed colocalization of GFP (green), GFAP (purple) and NeuN (red), representing a transitional stage in astrocyte to neuron conversion. Scale bar: 25 μm. **C**–**E** Representative western blot image and quantification analysis of the protein levels of DCX and NeuN at 14 dpi with or without ND1–GFP treatment. N = 6. ^#^*p* < 0.05 indicated comparison with sham group, *p < 0.05 and **p < 0.01 indicated comparison with SAH + GFP group

Whether NeuroD1 can induce astrocytes to neurons conversion in the hippocampus after SAH remains unclear. Immunofluorescent staining was conducted, and the results showed that ND1–GFP significantly increased the density of GFP^+^DCX^+^ cells than that in the GFP group at 14 dpi (Fig. 4A). Furthermore, mature neuronal marker NeuN was employed, and the immunofluorescent images showed that NeuroD1 induced significant conversion of GFAP^+^GFP^+^ into NeuN^+^GFP^+^ cells at both 7 dpi and 14 dpi (Fig. 4B), while few NeuN^+^GFP^+^ cells were identified in the GFP group. Interestingly, some ND1–GFP-infected cells were positive for both GFAP (magenta) and NeuN (red) as shown by the white arrowhead in Fig. 4B, which indicated a transitional stage during astrocytes to neurons conversion. Western blot again showed that the protein levels of both DCX and NeuN were significantly higher in the ND1–GFP group than that in the GFP group (*P* < 0.05, Fig. 4C–E). Collectively, these data indicated that NeuroD1 efficiently converted transfected cells, most likely astrocytes, into neurons at the early phase of SAH.

**Fig. 5:**
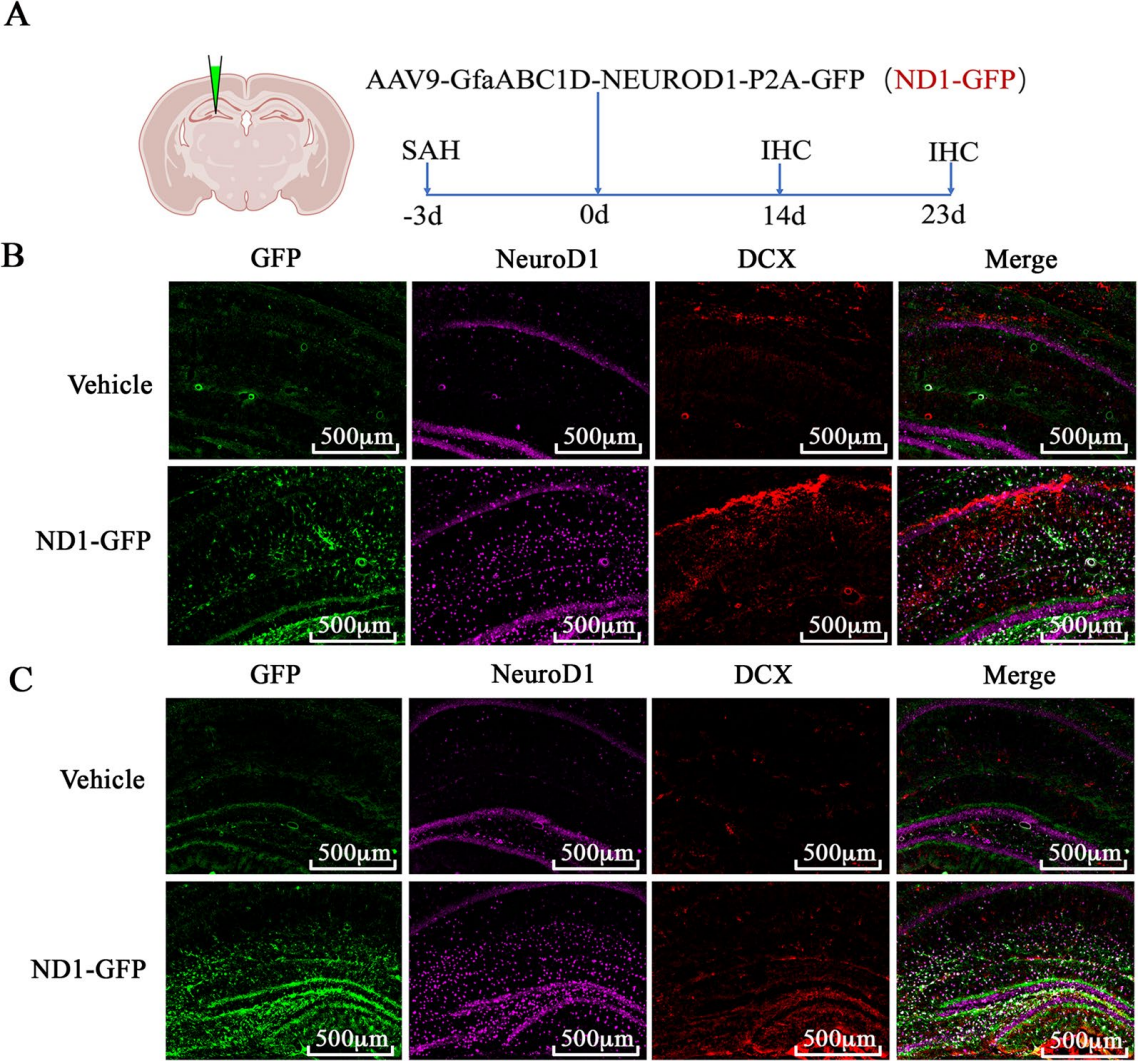
ND1–GFP-converted astrocytes to neurons at the early phase of SAH and boosted endogenous neurogenesis at the late phase of SAH. **A** Schematic illustration of the experimental protocol. **B** Representative immunofluorescent images showed that ectopic NeuroD1 mainly promoted the expression of DCX in the area away from SGZ at 14 dpi, indicating that neurogenesis mainly originated from ND1–GFP-mediated astrocytes reprogramming at 14 dpi. Scale bar: 500 μm. **C** Representative immunofluorescent images revealed that ectopic NeuroD1 mainly upregulated the expression of DCX at the region around SGZ at 23 dpi, indicating that neurogenesis mainly occurred from neural stem cells at 23 dpi. Scale bar: 500 μm

To understand how NeuroD1 impacted neurogenesis, we harvested the brain at 14 dpi and at 23 dpi, as illustrated in Fig. 5A. Compared to the control group, ND1–GFP significantly increased the expression of NeuroD1 throughout the hippocampus at 14 dpi and at 23 dpi (Fig. 5B, C). Again, the results showed that NeuroD1 significantly increased the number of DCX-labeled newborn neurons than that in the contralateral control side both at 14 dpi and at 23 dpi (Fig. 3B, C). Interestingly, at 14 dpi, immunofluorescent images showed that ectopic NeuroD1 mainly promoted the expression of DCX in area away from SGZ known for endogenous NSCs (Fig. 5B), indicating that DCX-labeled cells arose mainly from the in vivo reprogramming, but not endogenous neurogenesis. However, at 23 dpi, the expression of DCX mainly distributed at the region around SGZ (Fig. 5C), suggesting that NeuroD1 mainly boosted endogenous neurogenesis at the late phase of SAH. These results suggested that ectopic NeuroD1 mainly mediated astrocyte reprogramming at the early phase of SAH and restored endogenous neurogenesis at the late phase of SAH.

### NeuroD1 repaired the neural circuit and improved neurocognitive function after SAH

**Fig. 6:**
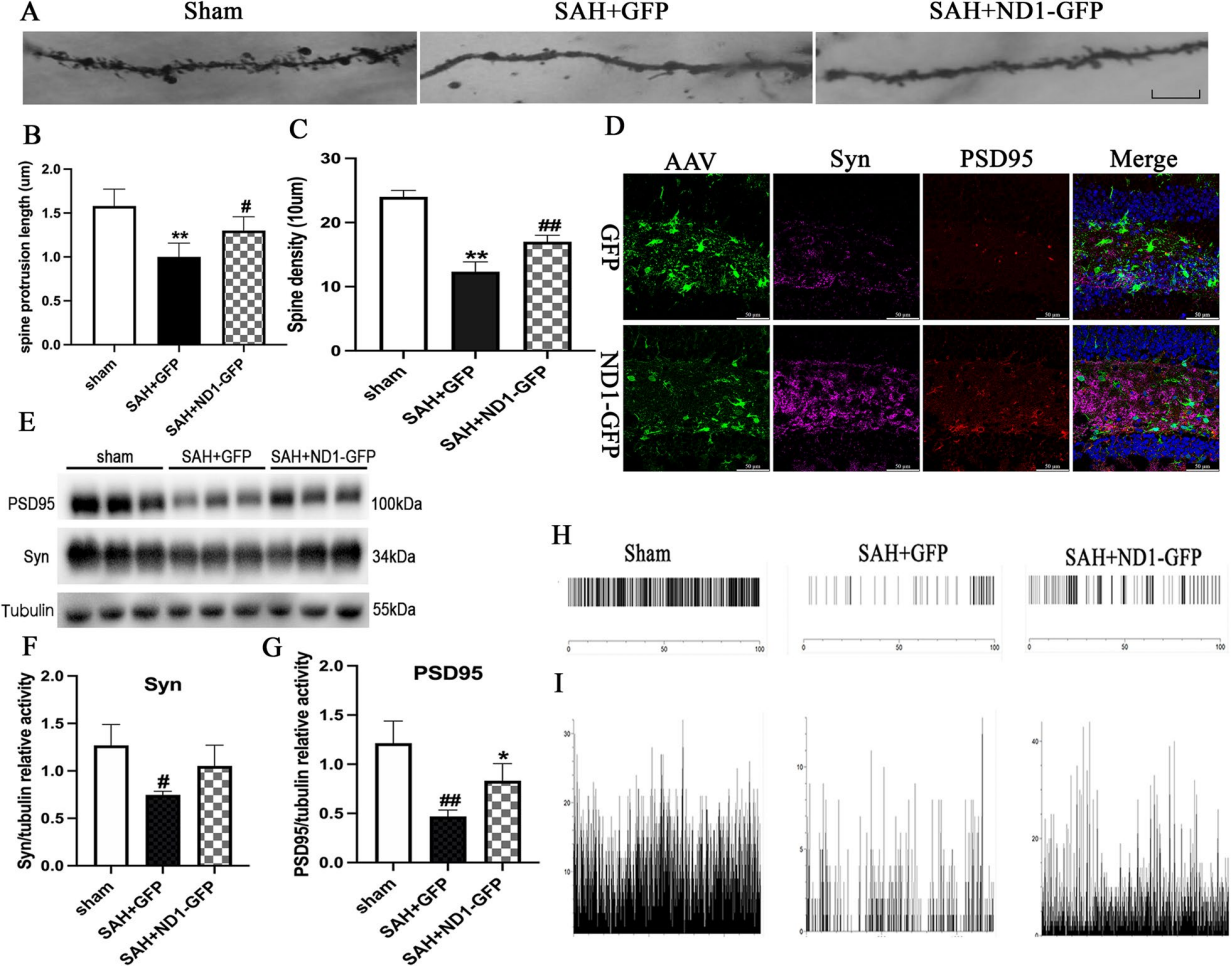
NeuroD1 treatment restored neural network following SAH. **A** Representative images of dendritic spines of in the hippocampus of mice injected with GFP or ND1–GFP. Scale bar, 10 μm. **B**, **C** Quantification analysis of the dendritic spine protrusion length and the spine density of neurons. *P < 0.05 and **P < 0.01 indicated comparison with Sham group; ^#^*P* < 0.05, ^##^*P* < 0.01 indicated comparison with SAH + GFP group; *n* = 6 per group. D. Representative immunofluorescent images showed the expression of PSD95 and Syn in the hippocampus at 14dpi. Scale bar: 50 μm. **E**–**G** Representative western blot image and quantification analysis of PSD95 and Syn showed that the protein levels of PSD95 and Syn were strongly increased after ND1–GFP injection. *N* = 6. ^#^*p* < 0.05, ^# #^*p* < 0.01 indicated comparison with sham group; *p < 0.05 indicated comparison with SAH + GFP group. **H**, **I** Firing rate and number of discharges of neurons in the electrophysiology in vivo were assessed at 14 dpi

Neuron to neuron synaptic connections form the neural circuit and are the cellular basis for brain function. Dendritic spine density is a representative for synaptic plasticity, which is closely related to learning and memory functions [19]. In this study, we found that SAH caused significant reduction in dendritic spine protrusion length and spine density. However, NeuroD1 treatment dramatically increased the dendritic spine protrusion length and the spine density than those in the GFP group (*P* < 0.05, Fig. 6A–C). Furthermore, the protein levels of the pre-synaptic marker Syn and the post-synaptic marker PSD95 were measured to assess the strength of synaptic connection in the hippocampus after SAH, immunofluorescent images showed that ND1–GFP treatment significantly increased the protein levels of both PSD95 and Syn than those in the GFP group (Fig. 6D), and western blot revealed that the protein levels of PSD95 and Syn were both significantly decreased after SAH induction (*P* < 0.05, Fig. 6E–G). However, NeuroD1 injection strongly increased the protein level of Syn than that in the GFP group, though the difference did not reach significance (*P* = 0.17, Fig. 6E–G). Moreover, NeuroD1 significantly increased the expression of PSD95 than that in the GFP group (*P* < 0.05, Fig. 6E–G), indicating that NeuroD1-induced neurogenesis significantly repaired the disrupted synapses after SAH.

To further determine whether the repaired synaptic connections by NeuroD1 was functional, we used neurophysiological recording to evaluate neural circuit function. The firing rate of neurons in the hippocampus were markedly impaired following SAH. However, NeuroD1 significantly increased the firing rate of neurons than that in the GFP group (Fig. 6H). Moreover, SAH induced dramatical decrease of the number of neuronal discharges, and NeuroD1 treatment significantly increased the number of neuronal discharge than that in the GFP group (Fig. 6I). Together, NeuroD1 efficiently repaired the neural circuit through mediating astrocyte to neuron conversion and restoring endogenous neurogenesis after SAH.

**Fig. 7:**
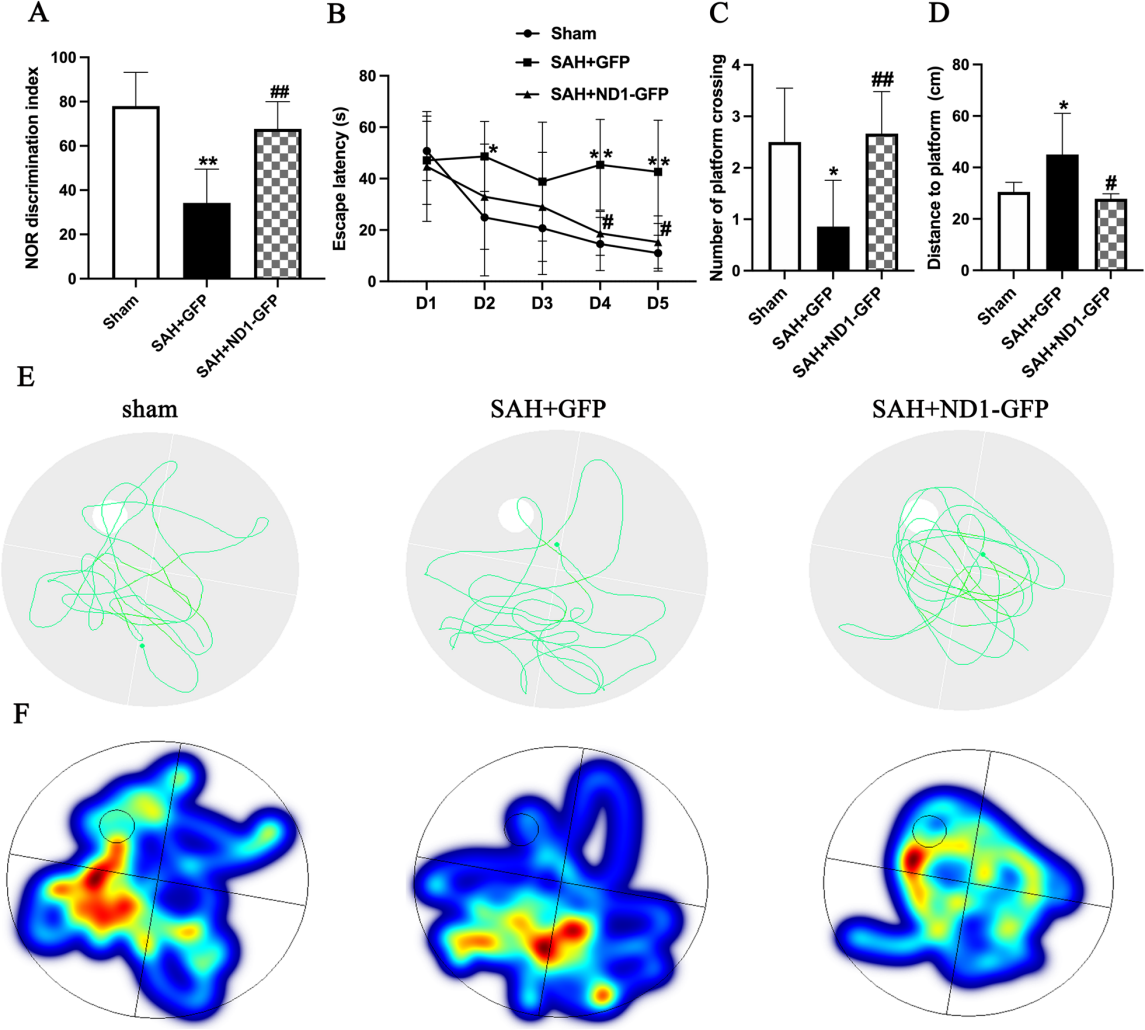
ND1–GFP significantly improved neurocognitive function after SAH. **A** NOR discrimination index was measured at 14 dpi. **B** Escape latency test in the water maze was performed from D1 (9 dpi) to D5 (13 dpi). **C** Number of platform crossings was calculated at 14 dpi. **D** Distance to the platform was assessed at 14 dpi. **E**, **F** Swimming path tracks and corresponding heat maps on the last day of water maze test. The mice in the SAH + ND1–GFP group were closer to the platform than the SAH + GFP group. *n* = 10, *P < 0.05 and **P < 0.01, SAH + GFP group vs sham group, ^#^*P* < 0.05 and ^##^*P* < 0.01, SAH + ND1–GFP group vs SAH + GFP group, by two-way ANOVA

Novel object recognition and Morris water maze were performed to assess the role of NeuroD1 on functional recovery following SAH. Novel object recognition was conducted at 11–14 dpi, and the results indicated that novel object recognition discrimination index was impaired after SAH, and NeuroD1 treatment significantly rescued the results than those in the GFP group (*P* < 0.05, Fig. 7A). Morris water maze was assessed at 9–14 dpi and the results showed that the number of platform crossing was significantly decreased and the distance to platform was markedly increased after SAH induction (*P* < 0.05, Fig. 7B–D. However, NeuroD1 treatment significantly increased the number of platform crossings than those in the GFP group, and the distance to platform was significantly shortened in the NeuroD1 group than that in the control group (*P* < 0.05, Fig. 7C–D, suggesting that injection of NeuroD1 markedly improved the neurocognitive function after SAH. After removal of platform at 14 dpi, swimming path tracks and heat maps showed that the memory of the mouse was significantly improved in the NeuroD1 group than that in the GFP group (*P* < 0.05, Fig. 7E, F).

## Discussion

**Fig. 8:**
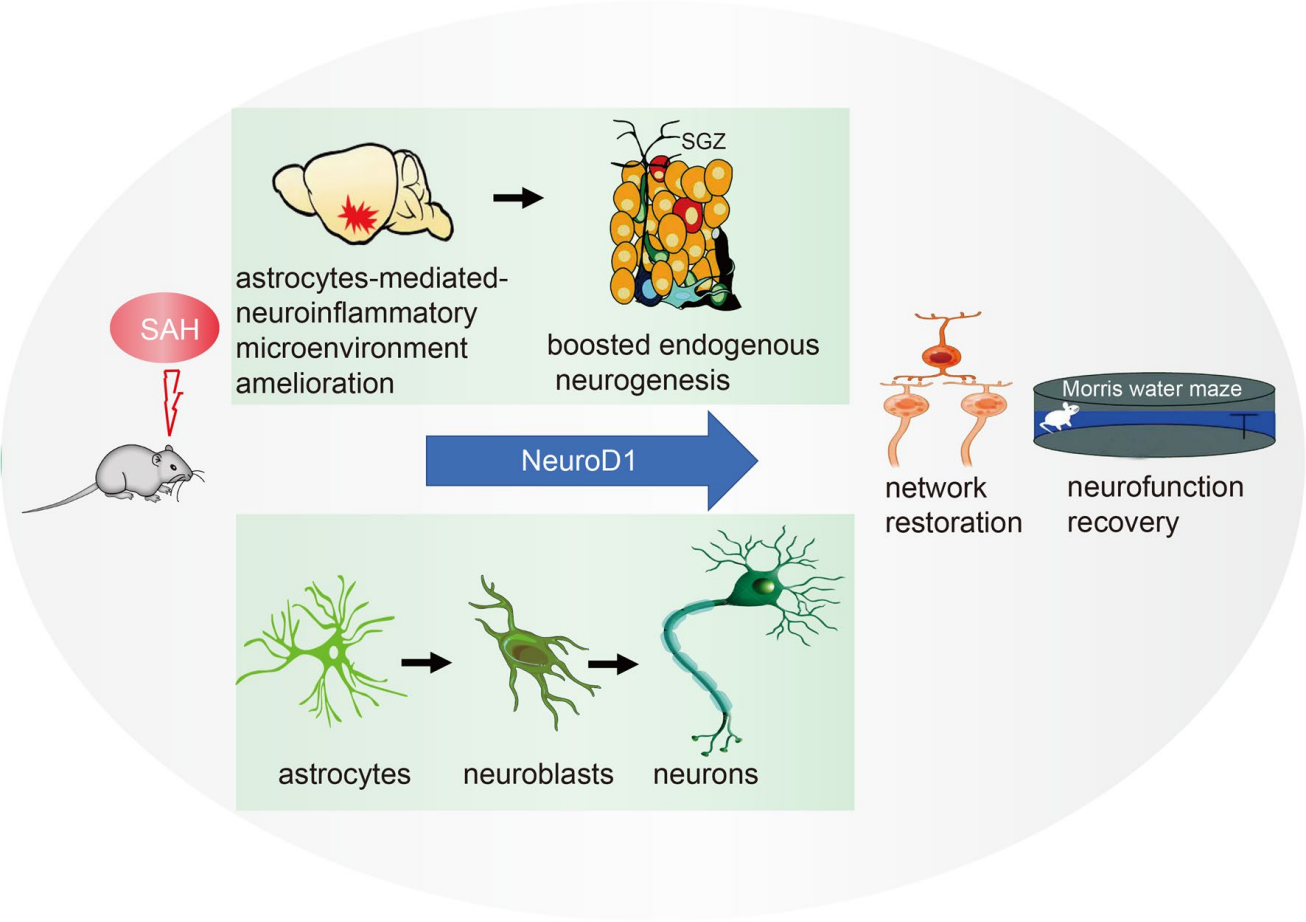
ND1–GFP treatment significantly improved neurocognitive function through transforming astrocytes to create a microenvironment that was friendly to boost endogenous neurogenesis and converting astrocytes to neurons following SAH

As illustrated in Fig. 8, we found that NeuroD1 significantly attenuated reactive astrocyte-mediated neuroinflammation and created a suitable microenvironment for neurogenesis following SAH. NeuroD1 efficiently converted transfected cells, most likely astrocytes, to neurons at the early phase of SAH. Likely benefit from the improvement of neuroinflammatory microenvironment, NeuroD1 administration significantly restored endogenous neurogenesis at the late phase of SAH. Functionally, NeuroD1 treatment promisingly restored neural circuit and improved neurocognitive dysfunction following SAH.

Reactive astrocytes and astrocytes proliferation are apparently induced by SAH [27]. As reported, some reactive astrocytes highly upregulated neuroinflammatory genes which may cause harm to neurons, and some other reactive astrocytes may provide trophic support for neurons [2]. Neurotoxic A1 astrocytes were significantly activated and were reported to mediate neuroinflammation following SAH [6]. Abundant A1 astrocytes secreted large amount of neuroinflammatory cytokines and stimulated harmful M1 microglia to aggravate the inflammatory microenvironment [28]. Furthermore, the neurotrophic factors, such as PTN, were significantly decreased and the BBB integrity were impaired, while the normal function of alternative A2 astrocytes was inhibited after brain injury [25, 29]. Therefore, it is important to restore the beneficial A2 astrocyte function and attenuate A1 astrocytes-mediated neuroinflammation following SAH.

In vivo reprogramming technology has gain wide attention in converting astrocyte to neuron [29, 30]. NeuroD1 is one of the most popular transcription factors which are suggested to convert astrocyte to neuron, although it remains debatable [14, 31]. Notably, previous studies mainly focused on the role of NeuroD1 in inducing astrocyte to neuron conversion, how NeuroD1 affected reactive astrocyte remained largely unknown. In this study, we found that NeuroD1 significantly attenuated astrocyte-mediated neuroinflammation, preserved BBB integrity and secreted neurotrophic factor PTN, which created a suitable microenvironment for neurogenesis. In addition, since reactive astrocyte can mediate neurotoxic M1 microglia activation [32], we also found that NeuroD1 significantly reduced the number of neurotoxic microglia following SAH. Likely benefited from the improvement of microenvironment, NeuroD1 significantly boosted endogenous neurogenesis at the late phase of SAH. Together, NeuroD1 significantly ameliorated reactive astrocyte-induced neuroinflammation and reboot endogenous neurogenesis following SAH.

NeuroD1 is a bHLH proneural transcription factor which plays an important role during embryonic brain development and adult neurogenesis [33]. In this study, we found that NeuroD1 efficiently converted transfected cells into neurons, and this reprogramming mainly occurred at the early phase of SAH. Interestingly, NeuroD1 also significantly boosted endogenous neurogenesis at the late phase of SAH. This may be because SAH-induced neuroinflammation hampered endogenous neurogenesis at the early stage, and NeuroD1 treatment significantly improved the microenvironment by transforming reactive A1 astrocytes and subsequentially reducing microglial activation, which together attenuated neuroinflammation and made the microenvironment suitable for endogenous neurogenesis at the late phase of SAH. In addition, as a neural transcription faction, NeuroD1 may boost endogenous neurogenesis by itself. However, the specific mechanism needs to be further investigated.

NeuroD1 treatment has been suggested to promote neurofunctional recovery in several brain diseases, although the mechanisms remained debatable [13, 31]. In this study, we found that NeuroD1 treatment significantly increased the synaptic density and upregulated the protein levels of Syn and PSD95 in the hippocampus following SAH. Synaptic connection is the physical basis for neuronal function, and the ability to form long-range axonal projections to their target regions and fire action potential is crucial for newborn neurons to integrate into the neural circuit [34]. In this study, electrophysiology examination revealed that NeuroD1 treatment significantly increased the firing rate of neurons and the frequency of neuronal discharge in the hippocampus following SAH. Moreover, the neurocognitive dysfunction after SAH was significantly improved by NeuroD1 treatment. Together, NeuroD1 treatment markedly promoted neurofunctional recovery by attenuating reactive astrocytes-mediated neuroinflammation and boosting neurogenesis following SAH.

There are some limitations in this study. First, since we focused on the role of NeuroD1 in attenuating reactive astrocytes-mediated neuroinflammation and boosting endogenous neurogenesis after SAH, we cannot exclude the possibility that NeuroD1 treatment also exerts other protective effects, such as the amelioration of neuronal death and preservation of autophagic function. Second, depending on only the immunofluorescent findings, we inferred that NeuroD1-converted astrocytes to neurons at the early phase of SAH. However, linage tracing technique is needed to confirm these findings, since whether the converted newborn neurons originated from neural stem cells, but not astrocytes, needs to be further determined.

## Conclusions

NeuroD1 significantly promoted neurofunctional recovery by attenuating astrocyte-mediated neuroinflammation and boosting endogenous neurogenesis following SAH. Specifically, NeuroD1 efficiently converted transfected cells into neurons at the early phase of SAH and boosted endogenous neurogenesis at the late phase of SAH, which likely benefited from the alleviation of neuroinflammatory microenvironment.

### Supplementary Information

**Additional file 1: Figure S1. **Representative photograph of the brain in the sham and SAH mice. **Figure S2.** PI-positive cells showed dramatical neuron death in the hippocampus at 3 days after SAH. Scale bar: 100 µm. **Figure S3.** GFAP-immunofluorescent image showed that astrocytes were highly activated at the hippocampus at 3 days after SAH. Scale bar: 100 µm. **Figure S4.** NeuroD1–AAV application significantly upregulated the expression of NeuroD1 in the hippocampus that has near completely succumbed to SAH. **Figure S5.** NeuroD1 expression ameliorated SAH-induced glial cell activation.

## Data Availability

The data sets used and/or analyzed during the current study are available from the corresponding author on reasonable request.

## Abbreviations

AHN: Adult hippocampal neurogenesis
BBB: Blood–brain barrier
DG: Dentate gyrus
Dpi: Day post injection
IACUC: Institutional Animal Care and Use Committee
NSCs: Neural stem/progenitor cells
PTN: Pleiotrophin
SAH: Subarachnoid hemorrhage
SGZ: Subgranular zone
Syn: A-synuclein
