# Supplementary material for: NeuroD1 administration ameliorated neuroinflammation and boosted neurogenesis in a mouse model of subarachnoid hemorrhage
Source: J Neuroinflammation. 2023 Nov 12;20:261. doi: 10.1186/s12974-023-02949-w (PMC10641988; doi:10.1186/s12974-023-02949-w)
Supplement: Supplementary file 1 — Additional file 1: Figure S1. Representative photograph of the brain in the sham and SAH mice. Figure S2. PI-positive cells showed dramatical neuron death in the hippocampus at 3 days after SAH. Scale bar: 100 µm. Figure S3. GFAP-immunofluorescent image showed that astrocytes were highly activated at the hippocampus at 3 days after SAH. Scale bar: 100 µm. Figure S4. NeuroD1–AAV application significantly upregulated the expression of NeuroD1 in the hippocampus that has near completely succumbed to SAH. Figure S5. NeuroD1 expression ameliorated SAH-induced glial cell activation. [file 12974_2023_2949_MOESM1_ESM.docx]

**Additional file 1**

**Figures and Figure legends**

**
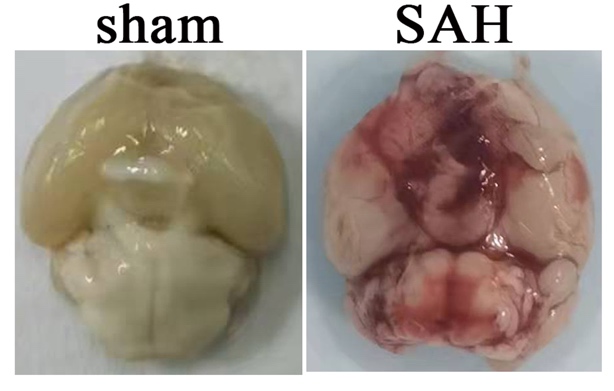
**

Figure S1. Representative photograph of the brain in the sham and SAH mice.


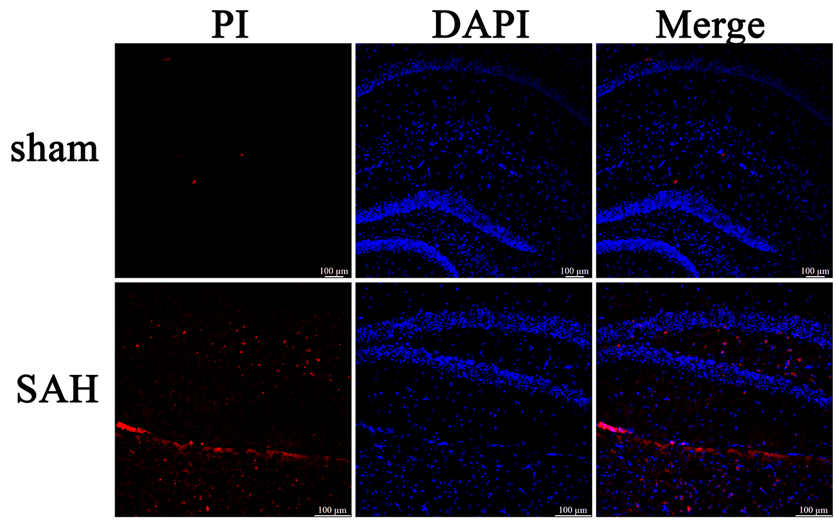


Figure S2. PI-positive cells showed dramatical neuron death in the hippocampus at 3 days after SAH. Scale bar: 100um.


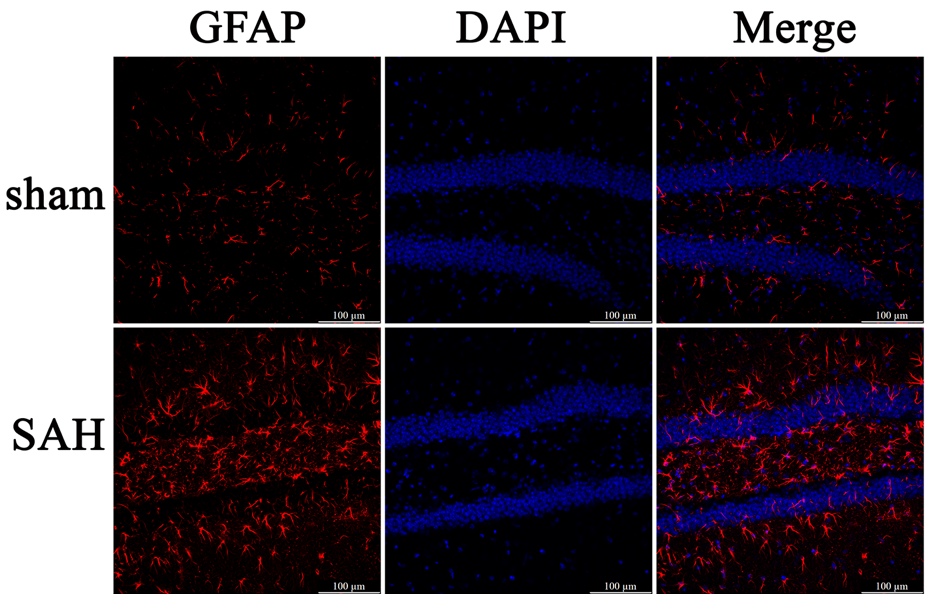


Figure S3. GFAP-immunofluorescent image showed that astrocytes were highly activated at the hippocampus at 3 days after SAH. Scale bar: 100um.


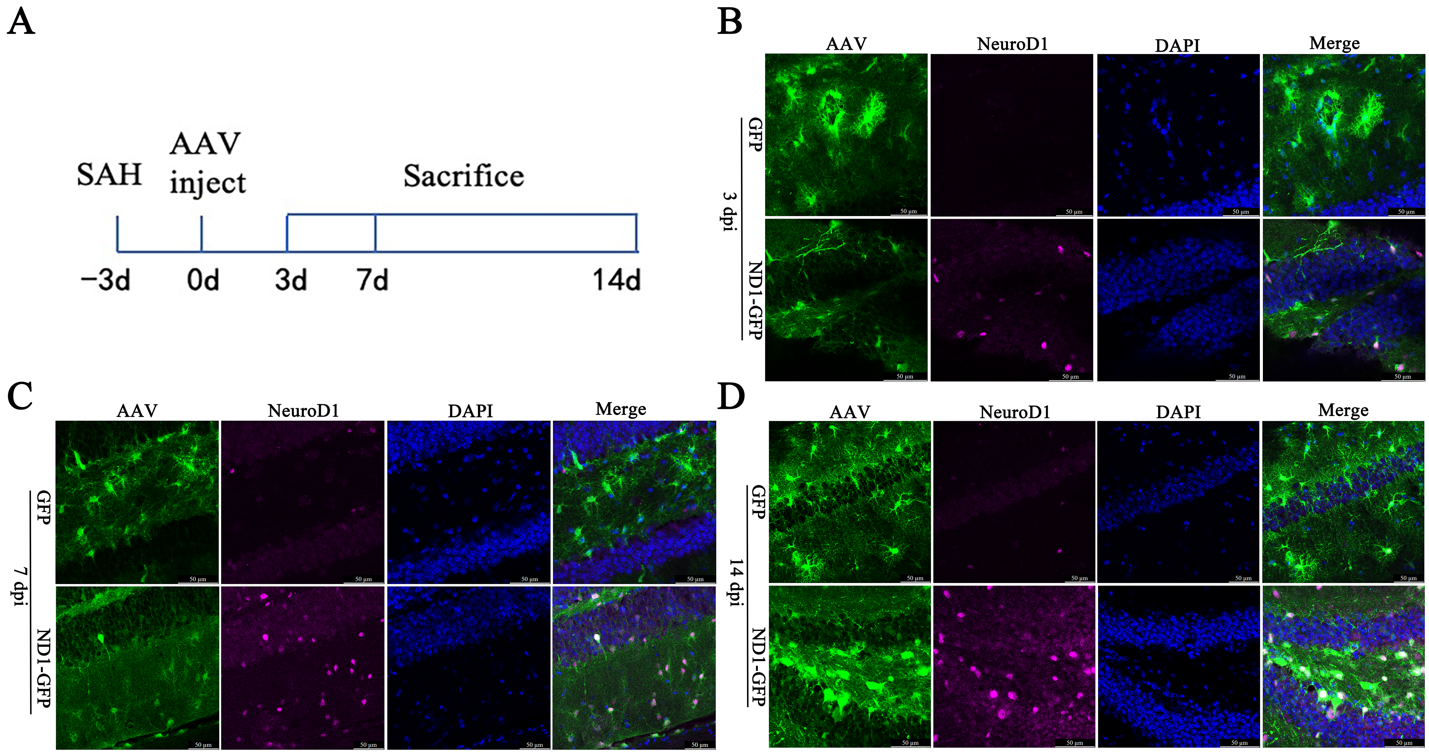


Figure S4. NeuroD1-AAV application significantly upregulated the expression of NeuroD1 in the hippocampus that has near completely succumbed to SAH. **(A).** Schematic illustration showed the experimental protocol of SAH induction, AAV injection and tissue harvest. **(B-D).** Immunofluorescent images indicated that NeuroD1-AAV significantly upregulated the expression of NeuroD1 in the hippocampus at different time points after SAH. Scale bar, 50um.


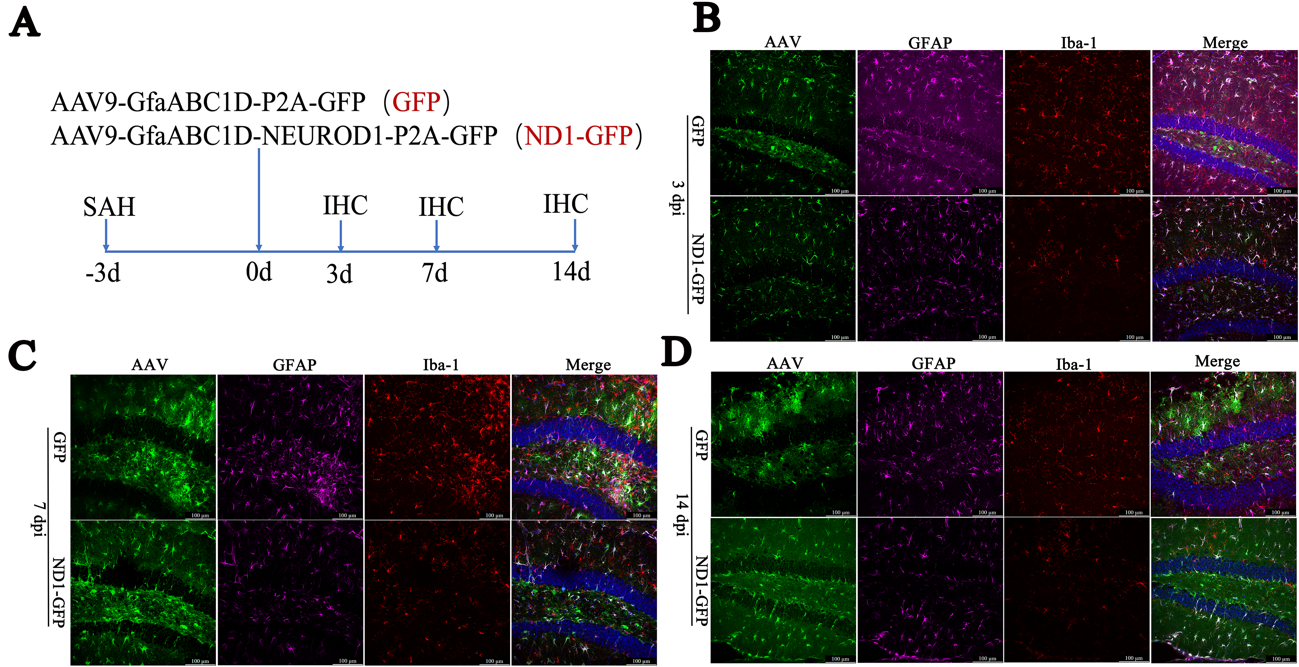


Figure S5. NeuroD1 expression ameliorated SAH-induced glial cell activation. **(A).** Schematic illustration of the experimental protocol. **(B-D).** Immunofluorescent images indicated that NeuroD1-AAV ameliorated the activation of astrocyte and microglia at relative time points after SAH. Scale bar: 100um.
